# Supplementary material for: Predictors of community pharmacists’ readiness to implement deprescribing of inappropriate medications for older adults in Qatar
Source: PLoS One. 2026 Feb 13;21(2):e0342795. doi: 10.1371/journal.pone.0342795 (PMC12904440; doi:10.1371/journal.pone.0342795)
Supplement: Attachment — Submitted filename: Response to Reviewers.docx [file pone.0342795.s002.docx]

9 July 2025

The Editor-In-Chief

PLOS ONE

Dear Sir,

Re: Manuscript #PONE- D-25-19691 – “Predictors of community pharmacists’ readiness to implement deprescribing of inappropriate medications for older adults in Qatar”

Our sincere thanks for the opportunity to revise the manuscript #PONE-D-25-19691, titled “Predictors of community pharmacists’ readiness to implement deprescribing of inappropriate medications for older adults in Qatar” which is under your consideration for publication in the PLOS ONE. We thank the reviewers for the insightful comments and useful suggestions and we have revised the manuscript accordingly. Please find stated below our point-by-point response to the reviewers’ comments. We have also revised the manuscript in accordance with the editor’s comments.

**EDITOR’S COMMENTS**

Journal Requirements:

1. When submitting your revision, we need you to address these additional requirements. Please ensure that your manuscript meets PLOS ONE's style requirements, including those for file naming. The PLOS ONE style templates can be found at https://journals.plos.org/plosone/s/file?id=wjVg/PLOSOne_formatting_sample_main_body.pdf and

https://journals.plos.org/plosone/s/file?id=ba62/PLOSOne_formatting_sample_title_authors_affiliations.pdf

**Response:** The corrections have been done in accordance with specifications stated in the PLOS ONE style template

2. Thank you for stating the following financial disclosure:

KBY

Undergraduate Research Experience Program (UREP) award [UREP29-092-3-029] from the Qatar National Research Fund (a member of The Qatar Foundation). The contents are solely the responsibility of the authors. Please state what role the funders took in the study. If the funders had no role, please state: "The funders had no role in study design, data collection and analysis, decision to publish, or preparation of the manuscript." If this statement is not correct you must amend it as needed. Please include this amended Role of Funder statement in your cover letter; we will change the online submission form on your behalf.

**Response:** The financial disclosure statement has been amended as recommended, and also included in the revised cover letter.

3. Please review your reference list to ensure that it is complete and correct. If you have cited papers that have been retracted, please include the rationale for doing so in the manuscript text, or remove these references and replace them with relevant current references. Any changes to the reference list should be mentioned in the rebuttal letter that accompanies your revised manuscript. If you need to cite a retracted article, indicate the article’s retracted status in the References list and also include a citation and full reference for the retraction notice.

**Response:** The references have been reviewed and revised as necessary.

**Additional Editor Comments:**

Introduction and literature review:

1. Please shorten this section, succinctly highlighting: the problem of polypharmacy, its prevalence in Qatar, the Middle East comparing to global patterns, why it needs to be addressed including any data relevant to Qatar e.g. prevalence of medicines related problems and how deprescribing can address them. Clearly defining deprescribing and how it can be done by community pharmacists. Many deprescribing interventions happen already, informally, over the counter in some countries. In others, deprescribing happens more formally following formal protocols and under specific job titles.

**Response:** The corrections have been done as recommended.

2. What are the barriers to community pharmacists deprescribing in Qatar and other countries.

**Response:** The manuscript has been revised to highlight the fact that the barriers have been assessed in a separate study that was previously published PLOS ONE.

3. Please justify the choice of theory.

**Response:** The manuscript has been revised to enhance the clarity of details of the justification for the choice of the theory.

Discussion

4. Please shorten this section be more succinct. Please discuss key findings (predictors of readiness to deprescribe and levels of knowledge) in relation to what was reported in other studies, taking care to succinctly highlight the relevant to Qatari context. It will be useful to highlight how this learning may feed into a future deprescribing service in community pharmacy in Qatar.

**Response:** The discussion has been revised accordingly.

5. It is noted that the pharmacy workforce is diverse, presented per nationality in the results section. What does this mean in relation to the findings e.g. practices pharmacists bring from their home country.

**Response:** The corrections have been done as recommended.

6. As a limitation it is important to address the limitations of answering questions about a service that is yet to exist.

**Response:** This has been addressed in the revised manuscript as recommended.

**REVIEWER’S COMMENTS**

**Reviewer #1:** Thank you for your submission of this research article on the knowledge, confidence and readiness of community pharmacists to implement deprescribing in Qatar which I really enjoyed reading and would be happy to see published pending minor revision **Response:** We are truly grateful for the valuable comments offered by the reviewer and the time spent to provide these useful feedback. We value the suggested corrections proposed by the reviewer and we are convinced it can only improve the scholarly value of the manuscript.

- **Comment-1**: You clearly highlighted the rationale for undertaking this research by using a theoretical framework and undertaking it in a developing country which does not yet have a community pharmacy deprescribing programme. Related to this (and the discussion on page 14 where it is suggested that results may differ by settings), consider briefly describing the scope of community pharmacists in Qatar for the international audience since this differs internationally i.e. Can they prescribe independently? Would they be able to stop/change medicines independently or would this be through a doctor?
- **Response-1:** We thank the reviewer for this excellent suggestion and we totally concur with the premise of the suggested correction and it has been done. [Pg 15, 1^st^ paragraph, line 4-7, 2^nd^ Paragraph, 12-18].
- **Comment-2:** The sample size calculation, questionnaire development/testing and statistical approaches (descriptive statistics of IQR/frequency and logistic regression) are all clearly described/justified.
- **Response-2:** We are truly grateful for the valuable comments offered by the reviewer and the time spent to provide these useful and kind feedback.
- **Comment-3:** The results of the three questionnaires included support the conclusion drawn that the majority of community pharmacists felt knowledgeable and confident. Related to these results, when Table 2 is described it is said that "However, a sizeable proportion of community pharmacists incorrectly described deprescribing as solely involving medication stoppage (42.5%, 85/200), or initiated only when adverse side effects are encountered by patients (28%; 56/200)" - however these frequencies combine those who answered incorrectly and those who answered don't know - hence rather than saying they incorrectly described deprescribing it would be more correct to say that they were "unable to correctly define"
- **Response-3**: This is an excellent suggestion and we sincerely thank the reviewer. The revision has been done as recommended [Pg 10, 1^st^ paragraph, line 8-11].
- **Comment-4:** The logistic regression included supports the conclusion drawn that female sex and exposure to deprescribing at undergraduate level were predictors of readiness to deprescribe. While the consideration of female sex in the literature is discussed, it is not for the other predictor - is there any relevant literature on the impact of deprescribing teaching in undergraduate pharmacy programmes that this could be linked to?
- **Response-4:** Heartfelt thanks to the reviewer for the valuable comment. The revision has been done as recommended [Pg 14, paragraph 2, line 15-20].
- **Comment-5:** The manuscript is clearly presented and readable but would benefit from editing as there are minor typographical and grammatical errors throughout - some examples include missing words like "a", changing from singular to plural within the same sentence (or vice versa) and some inconsistencies in Tables with missing brackets and sometimes within the same column using a decimal point for a whole number and other times not.
- **Response-5:** Heartfelt thanks to the reviewer for the suggestions. The corrections have been done as recommended.
