# Supplementary material for: Predictors of community pharmacists’ readiness to implement deprescribing of inappropriate medications for older adults in Qatar
Source: PLoS One. 2026 Feb 13;21(2):e0342795. doi: 10.1371/journal.pone.0342795 (PMC12904440; doi:10.1371/journal.pone.0342795)
Supplement: Attachment — Submitted filename: Response to Reviewers-2nd Rev.docx [file pone.0342795.s003.docx]

19 Nov 2025

The Editor-In-Chief

PLOS ONE

Dear Sir,

Re: Manuscript #PONE- D-25-19691_R1 – “Predictors of community pharmacists’ readiness to implement deprescribing of inappropriate medications for older adults in Qatar”

Our sincere thanks for the opportunity to revise the manuscript #PONE-D-25-19691_R1, titled “Predictors of community pharmacists’ readiness to implement deprescribing of inappropriate medications for older adults in Qatar” which is under your consideration for publication in the PLOS ONE. We thank the reviewers for the insightful comments and useful suggestions and we have revised the manuscript accordingly. Please find stated below our point-by-point response to the reviewers’ and editor’s comments. We have also revised the manuscript in accordance with the editor’s comments.

**EDITOR’S COMMENTS**

Journal Requirements:

1. If the reviewer comments include a recommendation to cite specific previously published works, please review and evaluate these publications to determine whether they are relevant and should be cited. There is no requirement to cite these works unless the editor has indicated otherwise.

**Response:** There was no such recommendation from the reviewers

2. Please review your reference list to ensure that it is complete and correct. If you have cited papers that have been retracted, please include the rationale for doing so in the manuscript text, or remove these references and replace them with relevant current references. Any changes to the reference list should be mentioned in the rebuttal letter that accompanies your revised manuscript. If you need to cite a retracted article, indicate the article’s retracted status in the References list and also include a citation and full reference for the retraction notice.

**Response:** The reference list has been reviewed and the suggested corrections have been done. There was no incidence of retraction.

3. In the manuscript, you reported that the questionnaire was informed by the situational theory of leadership; however, this was not clearly reflected in the questionnaire development section.

**Response:** The manuscript has been revised accordingly [Pg 6, 3^rd^ para, line 1-3].

4. You also indicated that the sampling technique used was purposive sampling. From your description, it appears that participants may have been recruited primarily based on ease of access and availability. This approach is generally referred to as convenience sampling rather than purposive sampling. Please carefully revisit your Methods section to ensure that the sampling technique is accurately described. If you intended purposive sampling, please clarify the specific inclusion criteria and rationale that guided participant selection.

**Response:** The purposive sampling method was used and the method section has been revised to enhance the clarity of the procedure used for sampling as recommended [Pg 6, 2^nd^ para, line 1-6].

5. In the Results section, you have reported frequencies in the format (percentage, n/total sample size). For consistency and clarity, please revise the reporting style to the standard format (n = XX, percentage%) instead of (n/total sample size). For example, please use (n = 109, 54.5%) instead of (109/200, 54.5%).

**Response:** The result section has been revised as recommended [Pg 9-12].

**REVIEWER’S COMMENTS**

**Reviewer #1:** Thank you for submitting your revised manuscript which you have clearly put a lot of work into which has strengthened your paper further.

**Response:** We are truly grateful for the valuable comments offered by the reviewer and the time spent to provide these useful feedback. We value the suggested additional corrections and we are convinced it can only improve the scholarly value of the manuscript.

- **Comment-1**: Your response -1: We thank the reviewer for this excellent suggestion and we totally concur with the premise of the suggested correction and it has been done. [Pg 15, 1st paragraph, line 4-7, 2nd Paragraph, 12-18]. Unfortunately, didn't make it any clearer for me (or perhaps other international readers) what the scope of community pharmacists in Qatar is i.e. Can they prescribe independently? Would they be able to stop/change medicines independently or would this only be through a doctor? Please clarify.
- **Response-1:** We thank the reviewer for this suggestion. The manuscript has been revised to emphasize the fact that the scope of practice for community pharmacists does not currently include the provision of deprescribing service in Qatar. [Pg 15, 2^nd^ para, line 18-20].
- **Comment-2:** There are minor typographical and grammatical errors throughout still - again examples include missing words like "a" or "the", changing from singular to plural within the same sentence (or vice versa) and some inconsistencies in Tables etc but the manuscript is still easily readable/understandable. Specific ones I would suggest to change based on your submitted amendments are:
- Abstract Line 4 should be "A" theory as at the start of the sentence not "a" theory
- Abstract Line 7 - remove "a" before Qatar
- Introduction Paragraph 3 - not having two sequential sentences starting with "indeed"
- Table 1 CPD - None not to one decimal place like others
- Table 2 - Extra bracket in "True" header
- **Response-2:** We are truly grateful for these valuable comments offered by the reviewer and the time spent to provide these useful and kind feedback. All the recommended corrections have been done.
- **Comment-3:** References - Not all in same format - one example would be 35 has no year listed for a journal article and not all formatting guidelines have been followed since reference citations are requested to be in brackets in the text as described https://journals.plos.org/plosone/s/file?id=wjVg/PLOSOne_formatting_sample_main_body.pdf
- **Response-3**: This is an excellent suggestion and we sincerely thank the reviewer. The in-text citations and the reference list have been revised as recommended.

**Reviewer #2:** The article is really informative and the topic 'deprescribing' is interesting.
The manuscript covered the basic research structure. Honestly, I cant find anything to be corrected.
Well done for the research team.

**Response:** Heartfelt thanks for the valuable comments offered by the reviewer. We feel greatly encouraged, and for that we are truly grateful.
